# Anomalous left coronary artery arising from the pulmonary artery in an adult women

**DOI:** 10.1007/s12471-016-0882-y

**Published:** 2016-08-18

**Authors:** M. C. de Kleijn, S. H. H. Kuijpers, F. J. Meijboom

**Affiliations:** 1 Department of Cardiology, Catharina Hospital, Eindhoven, The Netherlands; 2 Department of Cardiology, Maxima Medical Center, Veldhoven, The Netherlands; 3 Department of Cardiology, University Medical Center Utrecht, Utrecht, The Netherlands

**Fig. 1:**
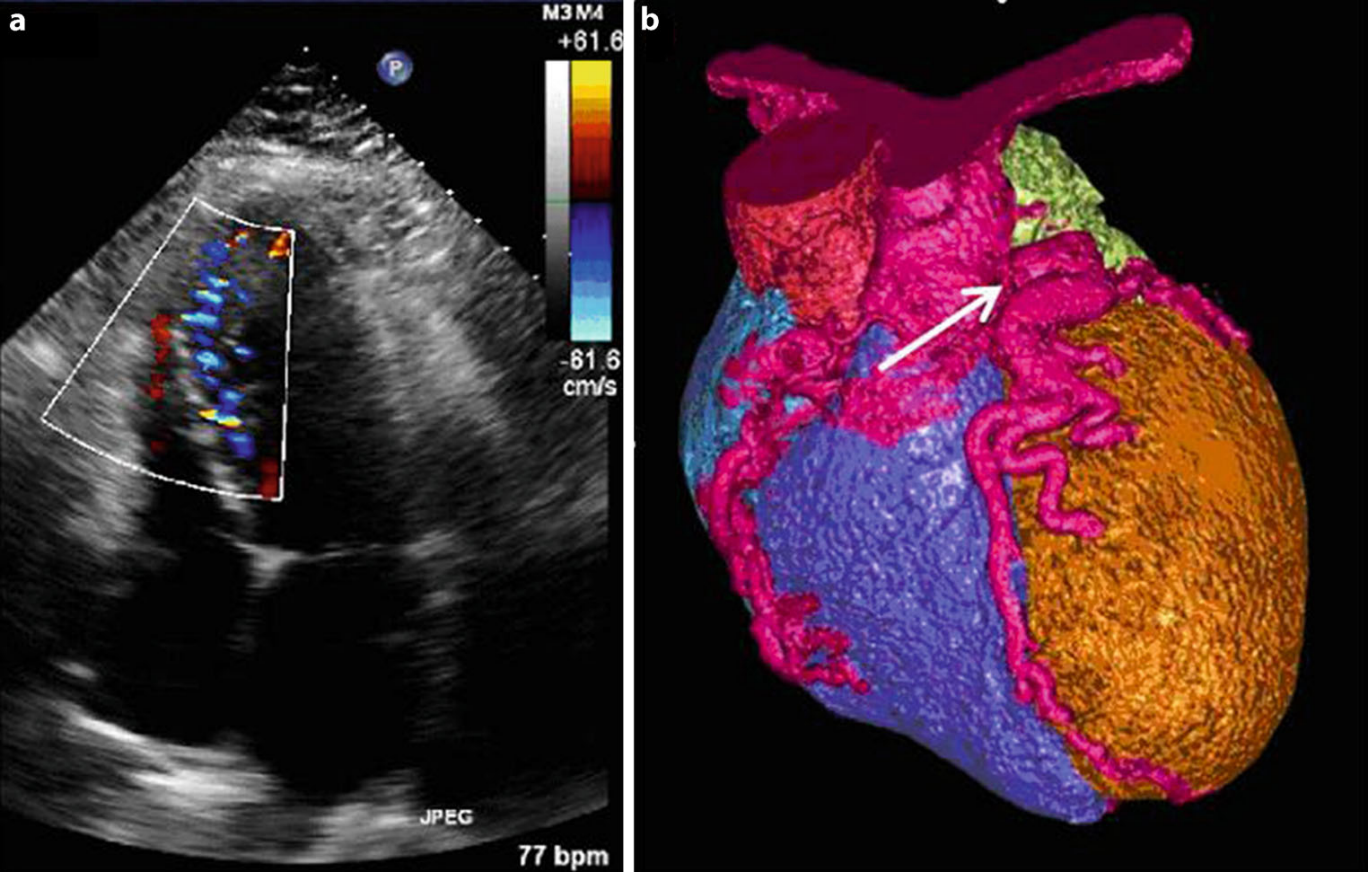
**a** Apical four chamber view showing extensive septal collaterals with colour Doppler.** b** Volume-rendered cardiac CT showing the left coronary artery originating from the pulmonary artery

A 47-year-old female was seen with chest pain and dyspnoea. She was diagnosed with an anomalous origin of the left coronary artery arising from the pulmonary artery or ALCAPA. This was previously known as the Bland-White-Garland syndrome. This congenital heart disease is a rare diagnosis in adults. Symptoms are angina, heart failure, ventricular arrhythmias, syncope or sudden death. Most patients are discovered in the first months of their lives, but patients can survive into adulthood because of an extremely large right coronary artery and an efficient network of collaterals [1, 2]. Specific echocardiographic changes are a large ostium of the right coronary artery and intercoronary septal collaterals (Fig. 1a; [3]). A 64-slice CT scan or MRI can be helpful in the diagnosis of ALCAPA (Fig. 1b). The treatment is re-implantation of the anomalous coronary into the aorta directly or with the help of a pulmonary flap. Treatment is necessary to prevent sudden cardiac death. [4, 5].
